# Real-world data reveal a diagnostic gap in non-alcoholic fatty liver disease

**DOI:** 10.1186/s12916-018-1103-x

**Published:** 2018-08-13

**Authors:** Myriam Alexander, A. Katrina Loomis, Jolyon Fairburn-Beech, Johan van der Lei, Talita Duarte-Salles, Daniel Prieto-Alhambra, David Ansell, Alessandro Pasqua, Francesco Lapi, Peter Rijnbeek, Mees Mosseveld, Paul Avillach, Peter Egger, Stuart Kendrick, Dawn M. Waterworth, Naveed Sattar, William Alazawi

**Affiliations:** 1 GlaxoSmithKline, London, UK; 2 Worldwide Research and Development, Pfizer, Connecticut, USA; 3 Erasmus Universitair Medisch Centrum, Rotterdam, The Netherlands; 4 Fundació Institut Universitari per a la Recerca a l’Atenció Primària de Salut Jordi Gol i Gurina, Barcelona, Spain; 5 Centre for Statistics in Medicine, NDORMS, University of Oxford, Oxford, UK; 6 Quintile IMS, London, UK; 7 Health Search, Italian College of General Practitioners and Primary Care, Florence, Italy; 8 Harvard Medical School, Harvard, Boston, MA USA; 9 University of Glasgow, BHF Glasgow Cardiovascular Research Centre, Glasgow, UK; 10 Barts Liver Centre, Blizard Institute, Queen Mary, University of London, London, UK

**Keywords:** Epidemiology, Population, NAFLD, NASH

## Abstract

**Background:**

Non-alcoholic fatty liver disease (NAFLD) is the most common cause of liver disease worldwide. It affects an estimated 20% of the general population, based on cohort studies of varying size and heterogeneous selection. However, the prevalence and incidence of recorded NAFLD diagnoses in unselected real-world health-care records is unknown. We harmonised health records from four major European territories and assessed age- and sex-specific point prevalence and incidence of NAFLD over the past decade.

**Methods:**

Data were extracted from The Health Improvement Network (UK), Health Search Database (Italy), Information System for Research in Primary Care (Spain) and Integrated Primary Care Information (Netherlands). Each database uses a different coding system. Prevalence and incidence estimates were pooled across databases by random-effects meta-analysis after a log-transformation.

**Results:**

Data were available for 17,669,973 adults, of which 176,114 had a recorded diagnosis of NAFLD. Pooled prevalence trebled from 0.60% in 2007 (95% confidence interval: 0.41–0.79) to 1.85% (0.91–2.79) in 2014. Incidence doubled from 1.32 (0.83–1.82) to 2.35 (1.29–3.40) per 1000 person-years. The FIB-4 non-invasive estimate of liver fibrosis could be calculated in 40.6% of patients, of whom 29.6–35.7% had indeterminate or high-risk scores.

**Conclusions:**

In the largest primary-care record study of its kind to date, rates of recorded NAFLD are much lower than expected suggesting under-diagnosis and under-recording. Despite this, we have identified rising incidence and prevalence of the diagnosis. Improved recognition of NAFLD may identify people who will benefit from risk factor modification or emerging therapies to prevent progression to cardiometabolic and hepatic complications.

**Electronic supplementary material:**

The online version of this article (10.1186/s12916-018-1103-x) contains supplementary material, which is available to authorized users.

## Background

Non-alcoholic fatty liver disease (NAFLD) is rapidly becoming the most common cause of chronic liver disease worldwide [1]. NAFLD is a spectrum of diseases that encompasses uncomplicated steatosis, non-alcoholic steatohepatitis (NASH) and fibrosis, which in a small proportion can lead to complications including cirrhosis, liver failure and hepatocellular carcinoma [2]. NAFLD is a multisystem disease with a multidirectional relationship with the metabolic syndrome [3–5]. NAFLD is associated with increased risk of cardiovascular disease [5–7] and cancer [8]. Among other high-risk groups [9], people with diabetes and NAFLD are at increased risk of micro- and macrovascular complications [10, 11] and these patients have a twofold increased risk of all-cause mortality [12].

The estimated point prevalence of NAFLD in the general Western population is 20–30%, largely based on cohort studies with heterogeneous inclusion criteria and research methods [13]. The prevalence of NAFLD rises to 40–70% among patients with type 2 diabetes and up to 90% among patients with morbid obesity [14–16]. Moreover, as the rates of diabetes and obesity rise worldwide, it is expected that NAFLD will become even more common. NAFLD-related cirrhosis is currently the third most common indication and is anticipated to become the leading indication for liver transplantation in the USA within the next one to two decades [17].

There is much debate about whether screening programmes in the general population or in at-risk groups, such as people with diabetes [9], should be implemented [18, 19]. This debate is based on our current understanding of the epidemiology and natural history of NAFLD, which, in turn, derives from cohort or cross-sectional studies [13]. These are often highly selected studies of individuals with metabolic risk factors, or they involve extensive phenotyping that would be unrealistic in routine practice.

A pragmatic approach is to focus on real-world patients for whom the diagnosis of NAFLD has been made during routine clinical care. A diagnosis of NAFLD is often made following abnormal imaging of the liver or elevated serum liver enzymes (so-called liver function tests) and involves exclusion of other causes of liver injury, such as excess alcohol consumption and viral hepatitis. Although routinely collected data can represent only the visible part of the clinical iceberg, there is a growing body of literature that has used well-curated electronic health records (EHRs) to study disease characteristics and epidemiology in large numbers of people [20–22].

In many European countries where health care is largely state funded and there are low or absent primary-care co-payments, the population has unrestricted access to health care with primary-care physicians acting as gatekeepers (including referral to secondary care) [23]. Healthy people register with primary-care centres when they move to an area to access health care when it is be needed and so primary-care EHR represent data that are as close to a general population as possible, with near universal coverage of the population in the region where the data is collected. Recording of a diagnosis in European primary-care databases is not driven by reimbursement and the patient population is relatively stable compared to other types of EHRs, such as US claims databases. Primary-care databases hold comprehensive medical records, which include diagnoses, prescriptions, laboratory values, lifestyle and health measures, and demographic information for a large and representative sample of patients. Concerns around the degree of data completeness are now largely historic as the vast majority of practices are paper-free and therefore, these data represent the only clinical record for care, administration and re-imbursement. Thus, within the areas that utilise these databases, coverage is near universal. If a practice joins the database, all the patients of that practice are registered in the database. Although there is an option for individual patients to opt out, this is minimal (<1%).

In this study, we harmonised health-care records for 17.7 million adults from four large European primary-health-care databases to estimate the prevalence and incidence of recorded diagnoses of NAFLD and, where available, NASH, in patients in primary care and to compare these with estimates from cohort studies. We sought to ascertain the changes in prevalence and incidence of recorded diagnoses of NAFLD from 2007 to 2015, and the effect of age and sex. We compared the characteristics of patients with an NAFLD diagnosis in the different databases and reported, where possible, the proportion of patients with markers of advanced disease in the diagnosed population.

## Methods

### Databases

Ethical approval was obtained by data custodians of each primary-care database according to local institutional review board requirements. Anonymised data were extracted from the Health Search Database (HSD) in Italy [24], the Integrated Primary Care Information (IPCI) in the Netherlands [25], The Health Improvement Network (THIN) in the UK [26] and the Information System for Research in Primary Care (SIDIAP) in the Catalonia region of Spain [27] (Additional file 1: Table S1).

THIN, HSD and IPCI had all reached high levels of patient registration from January 2004 onwards. SIDIAP started data collection in 2005 and has high quality data from 2006. Data entered between 1 January 2004 (SIDIAP from 1 January 2007) and up to 31 December 2015 were included in incidence estimates. Individuals were excluded if they had less than 1 year of follow-up post registration into the database. Individuals with a diagnosis of NAFLD were not included in the analyses if they also had a recorded history of alcohol abuse. To maximise data completeness, we included only patients whose NAFLD diagnosis occurred within ±6 months of a general practitioner (GP) visit when describing patients’ characteristics (Table 1 and Additional file 1: Table S3).

**Table 1:** Flow chart of identification of NAFLD patients

Flow chart	HSD (Italy)	IPCI (Netherlands)	SIDIAP (Spain)	THIN (UK)	Total
Total number of individuals ever enrolled by December 2015	1,571,651	2,225,925	5,488,397	12,695,046	21,981,019
Number of individuals with ≥1 year of registration in adulthood	1,544,573	1,780,500	5,259,575	9,085,325	17,669,973
Number of NAFLD patients	NAFLD: 27,002	NAFLD: 48,036 (19,048 were incident post IPCI starting date)	NAFLD: 77,547 NASH only: 1887	NAFLD: 23,529 NASH only: 1133	NAFLD: 176,114

The descriptive tables (Table 2 and Additional file 1: Table S3) include only patients with an incident diagnosis made within the study period and with a record of a GP visit within ±6 months of diagnosis. Numbers for NAFLD/NASH were as follows: HSD 24,027, IPCI 18,865, SIDIAP 77,107 and THIN: 12,385 individuals. Note in HSD and IPCI, ‘NAFLD’ is likely to include patients with NASH since no separate term for NASH exists in these databases. The number in the ‘Total’ column includes patients within SIDIAP and THIN who have NASH

*GP* general practitioner, *HSD* Health Search Database, *IPCI* Integrated Primary Care Information, *NAFLD* non-alcoholic fatty liver disease, *NASH* non-alcoholic steatohepatitis, *SIDIAP* Information System for Research in Primary Care, *THIN* The Health Improvement Network

### Patient involvement

All eligible patients were included in the study. Routine health-care records were collected from patients at each encounter with a health-care practitioner. Following local regulations, patients who did not wish to share their data were able to withdraw from the databases.

### Semantic harmonisation and case ascertainment

The four databases each use different coding systems (Additional file 1: Table S1). As a result, the capture of NAFLD and NASH diagnoses differed across databases. In HSD and IPCI, NAFLD and NASH were captured in a single code as ‘NAFLD or NASH’. In SIDIAP and THIN, NAFLD and NASH were coded separately, branching out of a ‘NAFLD or NASH’ code. In this study, we extracted all ‘NAFLD or NASH’ diagnoses as well as ‘NASH only diagnoses’ where available. For simplicity, we labelled ‘NAFLD or NASH’ as ‘NAFLD’ and ‘NASH only’ as ‘NASH’. Code lists were generated for the four terminologies (ICD9CM, Read Codes, SNOMEDCTUS and ICD10) that mapped to the same Unified Medical Language System (UMLS) concepts [28] (Additional file 1: Table S2).

Clinical diagnoses were defined with these code lists using the same process of harmonisation (code lists available on request). In SIDIAP, we used a combination of clinical codes and answers to questionnaires on alcohol consumption to identify alcohol abuse.

Given the absence of a code for NAFLD in the IPCI terminology, we additionally used text-mining in this database. The algorithm to identify NAFLD in IPCI is detailed in Additional file 1: Figure S1. Patients with records for the following search terms were extracted: ‘NASH’, ‘NAFLD’, ‘steatohepatitis’ or ‘fatty liver disease’ as distinct words preceded by a space and followed by a space, or at the beginning or end of a sentence. Patients with relevant search terms preceded by a negation term (e.g. ‘no’ or ‘not’) were excluded. To validate the text-mining, 100 individuals identified using free-text were randomly sampled. Their complete medical charts were manually reviewed to confirm that the clinical data support the text-mining-derived diagnosis.

#### Use of historical data

Governance rules differed between the different databases. In HSD and SIDIAP, there were no records available prior to a primary-care practice joining the database. In THIN, data from patients who had already left the practice were available, so NAFLD/NASH diagnoses made prior to the patient’s primary-care practice joining THIN were counted in both incidence and prevalence estimates. However, in IPCI, records that predated their primary-care practice joining the database were available only for patients who remained in the practice (since leavers did not have the opportunity to refuse to participate). Therefore, historic diagnoses could be included in the point prevalence. However, given that both the number of new diagnoses made as well as the total number of patients at risk in a given period were unknown, we could not include diagnoses made before the patient joined a practice in incidence estimates in IPCI.

#### Other data extraction

Demographic information, lifestyle and medical history of relevant morbidities were also extracted for all NAFLD and NASH patients identified in the four databases. Medical records for type 2 diabetes and hypertension at any time prior to NAFLD or NASH diagnosis were extracted. Code lists for those diagnoses were harmonised across the databases using the semantic harmonisation described in ‘Methods’, which aligns all terms for the same list of UMLS concepts (code lists available on request).

Laboratory values for aspartate transaminase (AST), alanine transaminase (ALT) and platelet count were extracted. We used the values closest to the time of NAFLD diagnosis (up to 2 years prior to diagnosis or less than 6 months after). Body mass index (BMI) was calculated for all NAFLD patients with weight recorded between 2 years prior to and 6 months after diagnosis, and with height recorded anytime in adulthood. We excluded values that were likely to be implausible: BMI below 15 kg/m^2^, laboratory values greater than the mean in the database plus 3 times the standard deviation, AST and ALT less than 5 IU/L, and platelet counts below 5 × 10^9^ L^–1^.

The FIB-4 index was calculated to provide an estimate of the severity of fibrosis in patients at the time of their NAFLD diagnosis. The formula for FIB-4 is: Age [years] × AST [U/L] / (platelet [10^9^] × √ALT [U/L]) [29]. The cut-offs for FIB-4 scoring for NAFLD are <1.30 for a low risk of advanced fibrosis or cirrhosis, between 1.30 and 2.67 for an indeterminate score and 2.67 for a high risk of advanced fibrosis or cirrhosis [30].

#### Statistical methods

Quantitative variables were reported as mean and 95% confidence interval (CI) of the mean assuming a normal distribution, and qualitative variables as percentages. Differences in patients’ characteristics between the four databases were tested by an ANOVA test for quantitative characteristics and a chi-square test for categorical characteristics.

Incidence in the adult population aged ≥18 years old was estimated by dividing the number of individuals with a diagnosis of NAFLD (or NASH where relevant) by the total number of person-years at risk. Incidence was reported by predefined age categories, gender and calendar year.

Point prevalence was estimated for 1 January of each calendar year available in the data, by gender and by predefined age categories. Point prevalence was defined as the total number of individuals with a recorded NAFLD diagnosis at or prior to 1 January of a calendar year and who were still active in the database, divided by the total number of active patients in the database on that date.

In addition, the 1-year period prevalence was estimated in a sensitivity analysis to account for potential differences in length of follow-up across databases, and over time within databases. The 1-year period prevalence was defined for each calendar year available as the number of new individuals with a recorded diagnosis of NAFLD in a calendar year divided by the average number of active patients in that year (defined as the number on 1 January plus the number on 31 December divided by 2).

Age was computed at the end of the year for period prevalence (31 December of the year of interest). For point prevalnce, age was computed on 1 January of the year of interest. Within each database, incidence estimates were compared by calendar year (assuming a linear relationship), sex (males are the reference group) and age group (age 60–69 is the reference group) by fitting Poisson distributions. Prevalence estimates were compared by fitting logistic regressions and performing chi-square tests. *P* < 0.001 was considered as significant, although note that with such large datasets, a high level of significance can be achieved even for minimal absolute differences in prevalence and incidence levels.

Incidence and prevalence estimates were pooled for each calendar year across the four databases using a random effects meta-analysis after natural log-transformation (weighting by the inverse of the variance). We reported the *I*^2^ statistic, which gives the percentage of variation among databases attributable to heterogeneity, and the *p* values of heterogeneity (p-het), tested using Q statistics. To investigate sources of heterogeneity, we tested for a linear association between incidence and point prevalence with calendar year by fitting a meta-regression.

Data were extracted and analysed using the European Medical Information Framework (EMIF) with a distributed network approach that allows data custodians to maintain control over their protected data [31]. Each data custodian extracted data from their database into four common files: prescriptions, measurements, events and patients. These files were transformed locally by the data transformation tool Jerboa Reloaded, which produces analytical datasets that can be shared with data analysts in a central remote research environment for further post-processing. The analytical datasets contained characteristics for each patient with a NAFLD diagnosis, as well as aggregated results on incidence and prevalence by age, gender and calendar year. Quality controls were run on each database and the research team communicated with data custodians to confirm results. Statistics and graphics were generated in the remote research environment using the statistical software Stata/SE 14.1.

## Results

### Semantic harmonisation to identify the European NAFLD cohort

In total, the four European databases held data on 21,981,019 patients, of whom 17,699,973 adults had been registered for at least 1 year in adulthood (Table 1). Using semantic harmonisation, we identified 176,114 patients who had a recorded diagnosis of NAFLD (including NASH). This represents 1.0% of the total population, ranging from 0.3% in the UK (THIN) to 2.7% in the Netherlands (IPCI). The largest number of NAFLD patients was in the Spanish cohort (SIDIAP, *n* = 77,547, Table 1). Recording of NASH diagnoses was possible only in Spain (SIDIAP, *n* = 1887) and in the UK (THIN, *n* = 1133), as the other two databases did not have specific codes distinguishing NAFLD from NASH. Given the small numbers overall, we did not pursue an analysis of NASH incidence and prevalence further and we included these patients within the total number of patients with a recorded diagnosis of NAFLD.

In the Dutch database (IPCI), the majority of patients were identified via free-text mining with seed words ‘NAFLD’, ‘NASH’, ‘fatty liver’ or ‘steatosis’, and a minority from diagnostic codes only (see Additional file 1: Figure S1). The code for ‘liver steatosis’ (D97.05) identified 1282 patients. The code for ‘cirrhosis/other liver disease’ (D97.00) identified 4228 patients when combined with a free-text search on the code label and 1214 additional patients when combined with a free-text search anywhere in the medical records. Searching for the search terms in free text in the absence of a relevant code identified 44,442 additional patients. Of these, 19,048 patients had an incident NAFLD diagnosis (recorded at a time when the patient’s general practice was contributing to IPCI). In the sample of 100 cases that were manually reviewed, the positive predictive value for a text-mined diagnosis of NAFLD was 98%.

We identified only a small proportion of patients with a recorded diagnosis of NAFLD who also drank alcohol in excess of recommended limits: 3130 (7.0%) NAFLD patients in IPCI, 921 in HSD (3.3%), 12,461 in SIDIAP (14.1%) and 925 in THIN (3.8%). These patients were excluded from the statistical analysis.

The characteristics of the populations of patients with an incident diagnosis of NAFLD made during the study period, after exclusions, are shown in Table 2 for the individual databases. There were minor differences in the mean age, proportion of patients with impaired fasting glucose or diabetes, and platelet count in each of the four databases. However, we observed that HSD had statistically significantly higher proportions of males and patients with hypertension than other databases. There was considerable variation in recorded BMI (29.7 kg/m^2^ in HSD to 32.4 kg/m^2^ in THIN), alanine transaminase (ALT) levels (median 28 IU/L in HSD to 39 IU/L in THIN) and aspartate transaminase (AST) levels (median 24 IU/L in HSD to 32 IU/L in THIN). Moreover, we observed variation in clinical practice with higher rates of BMI being recorded and ALT requests in THIN and SIDIAP compared to IPCI and HSD (Table 2 and Additional file 1: Table S3).

**Table 2:** Descriptive characteristics of patients with an incident diagnosis of NAFLD in four European primary-care databases

Characteristics	HSD (*N* = 24,027)	IPCI (*N* = 18,865)	SIDIAP (*N* = 77,107)	THIN (*N* = 23,385)	Test of difference *p* value
	% / Mean (SD) / median (IQR)	% / Mean (SD) / median (IQR)	% / Mean (SD) / median (IQR)	% / Mean (SD) / median (IQR)	
Age in years	56.2 (14.3)	56.8 (13.9)	56.0 (13.4)	53.7 (13.4)	<0.0001
Gender (males)	57.3%	49.3%	52.7%	51.5%	<0.0001
Body mass index in kg/m^2^	29.7 (5.0)	30.8 (5.4)	31.3 (5.1)	32.4 (5.9)	<0.0001
History of diabetes or impaired fasting glucose	18.0%	20.5%	20.0%	21.0%	<0.0001
History of hypertension	47.5%	36.0%	42.8%	40.5%	<0.0001
Aspartate transaminase (IU/L)	24 (19–33)	29 (22–40)	29 (22–40)	32 (24–47)	<0.0001
Alanine transaminase (IU/L)	30 (20–48)	37 (25–56)	34 (22–53)	45 (28–68)	<0.0001
Platelet counts (10^9^/L)	238 (65)	262 (68.6)	244 (61.2)	250 (75.3)	<0.0001
AST to ALT ratio	0.87 (0.34)	0.80 (0.36)	0.83 (0.38)	0.82 (0.38)	<0.0001
FIB-4 score					<0.0001
Low risk (FIB-4 < 1.30)	64.3%	70.4%	65.5%	65.0%	
Indeterminate risk (FIB-4: 1.30–2.67)	31.4%	26.7%	30.3%	25.0%	
High risk (FIB-4 > 2.67)	4.3%	2.9%	4.2%	10.0%	

Arithmetic means were reported for age, BMI, platelet counts and AST to ALT ratio; median (IQR) were reported for albumin, AST and ALT. *P* values are from ANOVA test of difference between means for continuous variables (for log-transformed AST and ALT), and Chi-2 test of difference for categorical variables. Number of patients with data available on each of these variables is provided in Additional file 1: Table S3

*ALT* alanine transaminase, *ANOVA* analysis of variance, *AST* aspartate transaminase, *BMI* body mass index, *HSD* Health Search Database, *IPCI* Integrated Primary Care Information, *IQR* interquartile range, *SD* standard deviation, *SIDIAP* Information System for Research in Primary Care, *THIN* The Health Improvement Network

Non-invasive scores that estimate the degree of liver fibrosis can be calculated from clinical parameters and are used to risk-stratify patients with NAFLD. Although both ALT and AST are required to calculate the majority of such non-invasive scores, ALT was more frequently available than AST in all four databases (Additional file 1: Table S3). An AST result was available for 21% (THIN) to 68% (HSD) and an ALT result for 67% (IPCI) to 86% (SIDIAP). This is reflected in the proportion of patients in whom a FIB-4 non-invasive assessment of liver fibrosis could be calculated, ranging from 11% in THIN to 54% in SIDIAP. Despite having the smallest number (and percentage) of patients in whom we could calculate FIB-4, the THIN database had the highest proportion of patients with high-risk scores indicative of advanced fibrosis or even cirrhosis (10.0% vs 2.9–4.3%, *p* < 0.001). In practice, patients with indeterminate or high-risk scores are often managed with further assessment leading to a liver biopsy. The proportion of patients with intermediate/high-risk scores was lower in IPCI (29.8%) compared to the other databases (35.0–35.7%); albeit the number of people for whom we could calculate FIB-4 was variable.

### The rising prevalence of NAFLD diagnosis

**Fig. 1:**
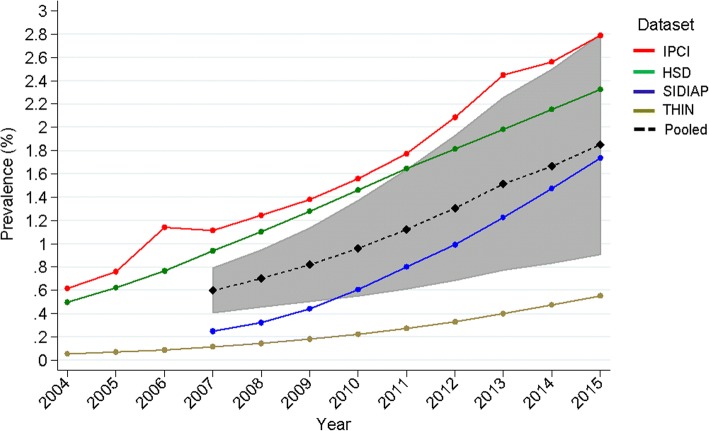
Point prevalence of NAFLD (per 100 persons) by calendar year. Results are shown for each database and pooled across databases by meta-analysis. The pooled estimate is provided from 2007 only as data from SIDIAP were available only from that year onward. The pooled estimate confidence interval is shaded grey. HSD Health Search Database, IPCI Integrated Primary Care Information, NAFLD non-alcoholic fatty liver disease, SIDIAP Information System for Research in Primary Care, THIN The Health Improvement Network

The overall (pooled) prevalence of NAFLD diagnosis was low at 1.85% (95% CI: 0.91–2.79) (*I*^2^ = 99.99%, p-het < 0.001) on 1 January 2015, but it had trebled from 0.60% (0.41–0.79) (*I*^2^ = 99.97%, p-het < 0.001) on 1 January 2007 (Fig. 1 and Additional file 1: Table S4).

The prevalence of recorded NAFLD diagnosis rose over time in all databases, albeit levels and rates of rise differed between databases, being highest in the Netherlands (IPCI) and lowest in the UK (THIN). To confirm that those trends were not due to more complete medical records being available in more recent years, we also estimated 1-year period prevalence and observed rising trends for the four databases (Additional file 1: Table S5).

**Fig. 2:**
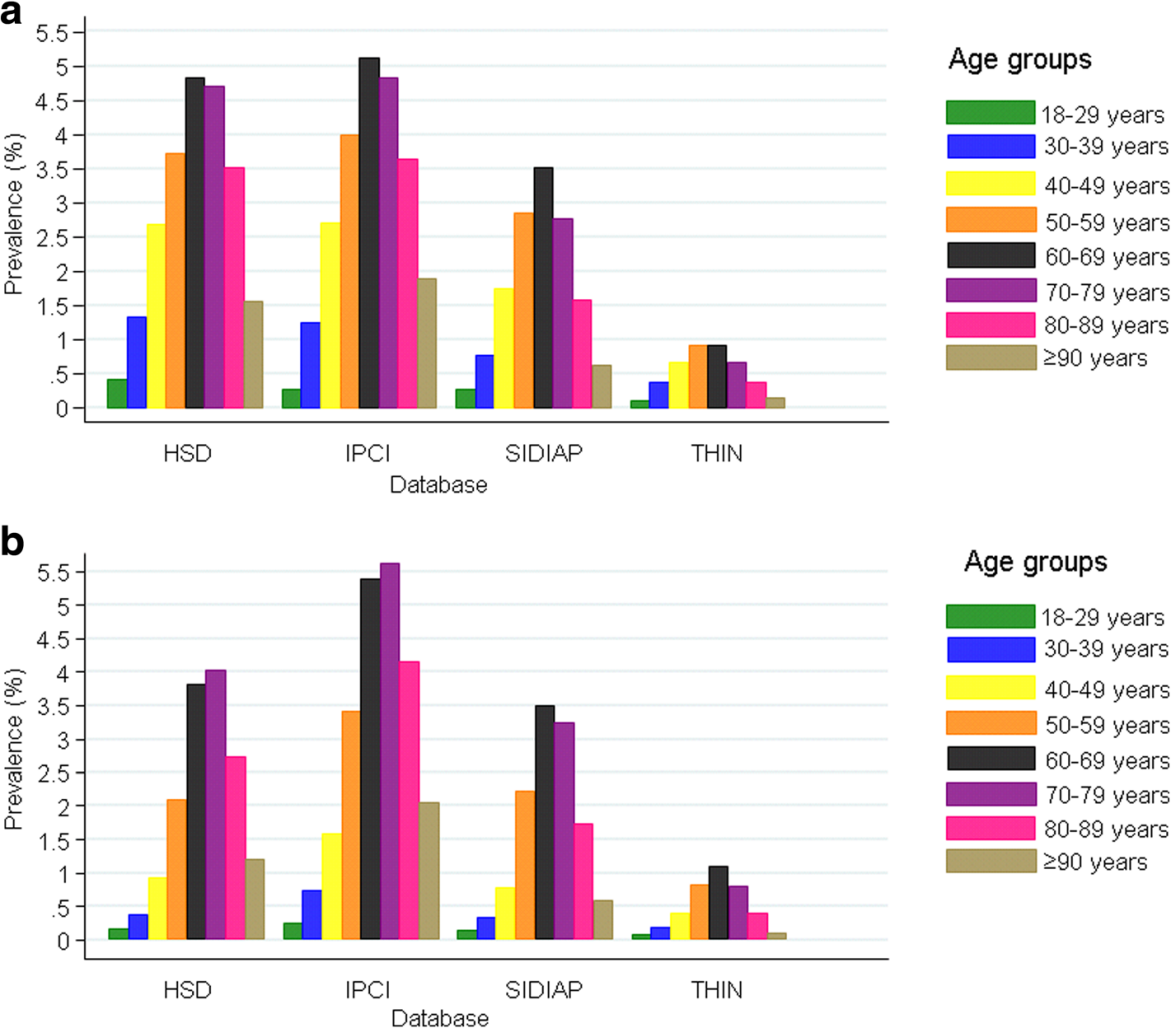
Point prevalence of NAFLD (per 100 persons) by age group on 1 January 2015 in **a** males and **b** females. HSD Health Search Database, IPCI Integrated Primary Care Information, NAFLD non-alcoholic fatty liver disease, SIDIAP Information System for Research in Primary Care, THIN The Health Improvement Network

There were no significant differences in prevalence between sexes in any database, but prevalence did vary by age. Peak prevalence was in patients aged 60–79 in whom it was >20 times higher than in 18–29 years old in IPCI (4.89% versus 0.24%) and 10–14 times higher in the other databases (Fig. 2 and Additional file 1: Table S6).

### Incidence of NAFLD has doubled since 2007

**Fig. 3:**
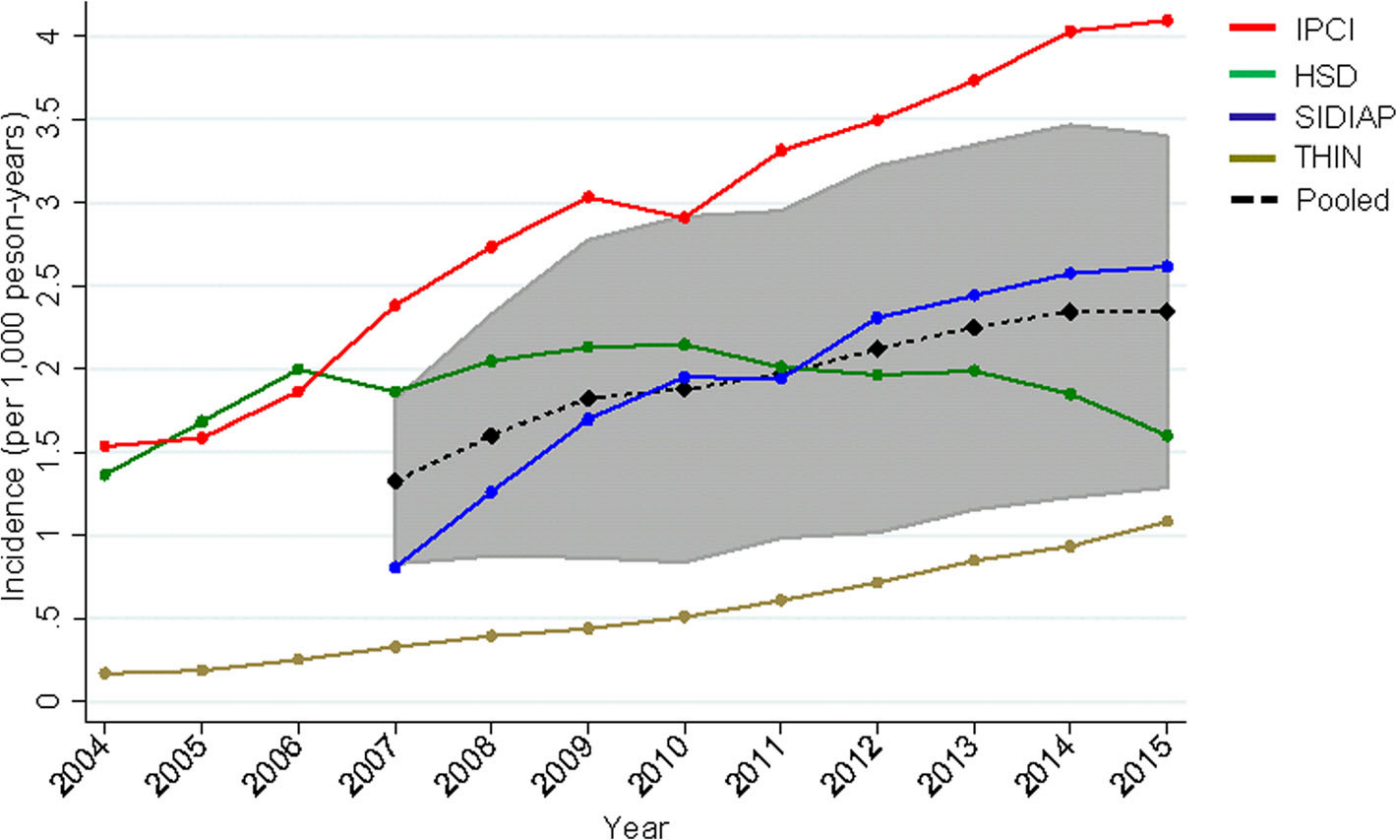
Incidence of NAFLD (per 1000 person-years) by calendar year in four primary-care databases, and pooled across databases by a random effects meta-analysis. The pooled estimate is provided from 2007 only as data from SIDIAP were available only from that year onward. The pooled estimate confidence interval is shaded grey. HSD Health Search Database, IPCI Integrated Primary Care Information, NAFLD non-alcoholic fatty liver disease, SIDIAP Information System for Research in Primary Care, THIN The Health Improvement Network

The overall (pooled) incidence of recorded NAFLD diagnoses was 2.35 (1.29–3.40; *I*^2^ = 99.92%, p-het < 0.001) per 1000 person-years in 2015, having approximately doubled since 2007 (1.32; 0.83–1.82)) (see Fig. 3 and Additional file 1: Table S7).

We observed heterogeneity between databases. In IPCI and SIDIAP, there was a clear and consistent rise in incidence with a 2.7-fold increase from 2004 to 2015 to 4.09 per 1000 person-years in IPCI and 3.2-fold increase from 2007 to 2015 to 2.61 per 1000 person-years in SIDIAP. In HSD, there was no statistically significant change in incidence between 2005 and 2015 (Additional file 1: Table S6). Although the rate of rise in THIN was comparable to IPCI and SIDIAP, the very low starting rate meant that despite a fivefold increase, the absolute increase was still modest and the incidence in 2014 was 1.08 per 1000 person-years.

**Fig. 4:**
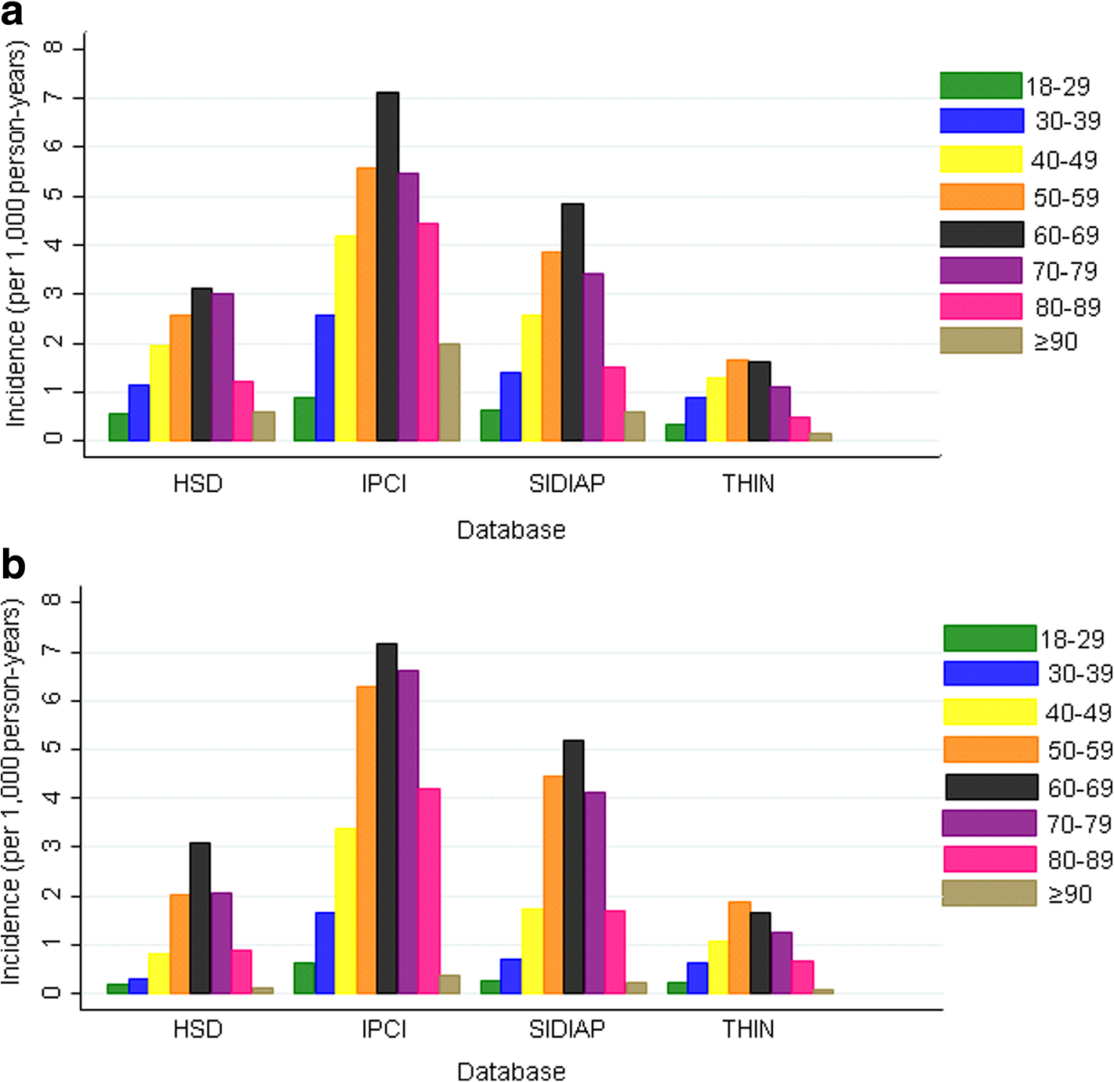
Incidence of NAFLD (per 1000 person-years) by age group for the four primary care databases for 2015 in **a** males and **b** females. HSD Health Search Database, IPCI Integrated Primary Care Information, NAFLD non-alcoholic fatty liver disease, SIDIAP Information System for Research in Primary Care, THIN The Health Improvement Network

There was a significant difference between sexes in HSD and SIDIAP (*p* < 0.05) but not in IPCI and THIN. In HSD, IPCI and SIDIAP, peak incidence was in 60–69 year olds, and in 50–59 year olds in THIN (but the estimate was not significantly different from that in 60–69 year olds) and then decreased in older age groups (Fig. 4, Additional file 1: Table S8).

## Discussion

In the largest real-world study of its kind to date, we report the incidence and prevalence of recorded NAFLD diagnoses among 17.7 million adults in four different European countries.

The databases used have been validated, are broadly representative of the population of the country and have been extensively used for pharmaco-epidemiology research [17, 20] (Additional file 1: Table S1). Despite a rise in incidence, our study found a large shortfall in Europe between the expected number of patients with NAFLD and NASH and the number with recorded diagnoses. Although others have suggested that this might be the case at a local level or in small questionnaire-based exercises [32], this study has identified the scale of that diagnostic gap across four European territories. Under-recording of NAFLD in primary care may reflect (i) missed opportunities to make the diagnosis by investigating abnormal liver enzyme values or imaging findings, (ii) a lack of confidence to make the diagnosis even if liver enzymes are in the reference range or (iii) under-recognition of the diagnosis in secondary care. Furthermore, many patients who do have the diagnosis have not had the investigations required for appropriate risk-stratification and therefore, specialist care may not be offered to those at greatest need. The current study represents a departure from existing population-level study designs of NAFLD. Notwithstanding the limitations discussed below, by using real-world data, we have gained insight into current practice and attitudes to NAFLD and into the changing face of NAFLD in primary care.

We used UMLS semantic harmonisation to extract primary-care EHR data and identify 176,114 patients with a recorded diagnosis of NAFLD. Despite variations in coding systems, in the characteristics of the populations and in the health-care systems in each country, the results from all four territories are broadly consistent. They show rising incidence and prevalence of NAFLD; however, the levels of recorded NAFLD in EHR primary-care databases is many-fold lower than those anticipated based on prior observation studies, which estimated the prevalence of NAFLD in the general European population to be 20–30% [33]. The characteristics of patients in that study were comparable with those with NAFLD in a recent systematic review of the literature and meta-analysis that included 101 studies [13]. That study reported the European prevalence of NAFLD diagnosed by imaging to be 24% (95% CI: 16–34%) and diagnosed by blood tests to be 13% (95% CI: 4–33%). Thus, our pooled prevalence in European EHR databases of 1.9% is at best ~1/6 and more likely only ~1/12 of the estimates based on cohort data. Our estimates of incidence in 2015 ranged from 1.1 to 4.1 per 1000 and are approximately 10 times lower than expected based on cohort studies: 28 (95% CI: 19–41) per 1000 person-years in Israel and 52 (95% CI: 28–97) per 1000 in Asia [13].

The prevalence of NAFLD diagnosis has trebled and incidence has doubled over the period of this study. The rising rates of co-morbid conditions such as diabetes and obesity may be responsible for this. Other probable factors include increased awareness among primary-care and non-liver physicians, improved communication of the diagnosis from secondary to primary care, and the increased use of blood tests and imaging to investigate common complaints such as abdominal pain or monitoring long-term conditions. Our data do not allow us to test these hypotheses further; however, studies from other groups also suggest that the total number of people developing NAFLD is rising, as is the number of people with NAFLD who develop life-threatening complications [13].

Despite the consistency in overall findings, the differences between the databases are indicative of differing practices. SIDIAP had a relatively large proportion of patients with a history of alcohol abuse (14.1%), although all databases included at least some NAFLD patients with recorded alcohol abuse. This reflects uncertainty in the community as to whether an individual can have fatty liver disease associated with metabolic syndrome even if they drink alcohol in excess of recommended limits, or indeed have any other cause of chronic liver injury such as viral hepatitis. While clinical trials make very precise distinctions between alcoholic and non-alcoholic fatty liver disease, the reality is that an obese, diabetic and hypertensive patient can consume alcohol in excess of recommended limits and have liver injury. There is no way to distinguish which aetiology is the dominant cause, and so clinicians are quite comfortable with co-existing diagnoses. Indeed, some authors now refer to BAFLD – both alcoholic and fatty liver disease. An alternative explanation may be that specialists making the diagnosis of fatty liver are unaware of the high alcohol use, either because of under-reporting by patients or poor communication from GP practices.

In HSD, prevalence increased over time whereas incidence has decreased in recent years. This can be explained by a relatively stable population in which nearly all patients were enrolled in 2000, see Additional file 1: Figure S3, and remained in the database until December 2015.

Text-mining in IPCI increased the number of NAFLD diagnoses by over eightfold. This suggests that while the diagnosis of NAFLD is being made, GPs are not recording it, despite there being a code for liver steatosis in IPCI. IPCI had the lowest level of ALT recording. A recent survey of Dutch GPs explored attitudes to the importance of NAFLD [34]. Only 47% of doctors used liver tests in patients with NAFLD and non-invasive scores were never used by 73% of respondents (we were able to calculate FIB-4 scores in only 27% in IPCI).

The UK THIN database appears to outlie from the others in several ways. The prevalence of recorded NAFLD in THIN (0.2%) is much lower than the other databases and markedly lower than that found in a study of almost 700,000 adults in a primary-care EHR study in London, UK (0.9%) [35]. Higher rates of alcohol recording in the UK alone are unlikely to account for all this difference. The median ALT was highest in THIN. This may suggest that the diagnosis of NAFLD is more likely to be made in the UK by investigating abnormal liver enzymes than in other territories. However, the data required to calculate FIB-4 were available in only 11% of patients in THIN (Additional file 1: Table S3). NAFLD patients in THIN had the highest mean BMI. Moreover, THIN had the highest proportion of NAFLD patients with diabetes or impaired fasting glucose and the highest proportion of NAFLD patients with high-risk FIB-4 scores. Large-scale liver-biopsy-based cross-sectional studies or replication of the current study in cohorts with systematic ascertainment of the component of FIB-4 would be needed to confirm that patients are diagnosed with NAFLD at more advanced stages in the UK compared to other European countries.

### Limitations of the study

When interpreting the data, it is important to consider the following issues. In IPCI, a diagnostic code for NAFLD was not available, therefore we devised an algorithm based on the diagnostic code ‘liver steatosis’ and excluding excess alcohol consumption. We did not do this for all databases because the IPCI terminology contains only 1073 clinical terms and therefore, general practitioners often utilise the free text to record information with greater precision, whereas the other coding systems contain many more such concepts: ICD9CM contains 40,855 terms, ICD10 contains 13,505 terms and Read Codes contains 347,568 terms [36].

The number of cases of recorded NASH is too small to make meaningful estimates of incidence and prevalence: 2–4% of patients with NAFLD in THIN and SIDIAP in which NASH was coded. This is far short of the 12.2% estimated from a US biopsy-based study [37]. This shortfall between coded NASH and the true burden of disease is probably due to the same factors that result in under-recording of NAFLD diagnosis: recognition, referral and coding in primary care, and under-diagnosis or poor communication in secondary care.

It is not possible to verify the accuracy or origin of recorded diagnoses, although the characteristics of the patients derived from the four databases are in keeping with the population one would expect with a NAFLD diagnosis. Some individuals not in this study may have undiagnosed NAFLD. Therefore, our results do not represent the true disease burden in the epidemiological sense, rather they tell us what is actually happening with people who currently have a diagnosis of NAFLD and they can inform the arguments for or against greater action in this area. While we cannot exclude the possibility (however unlikely) that all the other millions of expected NAFLD patients exist in other databases, we do not make any conclusions about people outside this dataset. Although primary-care data contain a large body of information, this does not diminish the value of well-phenotyped cohort studies in which NAFLD can be ascertained systematically using standardised screening methods (e.g. measuring liver enzymes or performing ultrasound in all patients). That said, the databases included in this study have been extensively used for research and have been validated for diagnoses other than NAFLD [24, 27, 38].

## Conclusions

Clinical practice is evolving in this emerging field and as yet there are no recommendations to screen formally for NAFLD, even in high risk groups [39, 40]. One school of thought is that if the only available intervention for NAFLD or NASH is lifestyle change, then doctors are already giving such advice to their patients, although the extent to which patients take up such advice varies. However, hepatic steatosis is an independent predictor of diabetes [41, 42] and could, therefore, identify patients who stand to benefit from lifestyle changes to prevent diabetes and hepatic complications. Furthermore, the emerging data suggesting hepatic steatosis is an independent cardiovascular risk factor may be an additional incentive for physicians to increase their awareness of the early stages of NAFLD. At the more severe end of the scale, novel therapies targeted at NASH and fibrosis are already in phase III clinical trials and are expected to be available in the next few years. These may change the treatment paradigm. Therefore, the scale of the health-care challenge posed by NAFLD and its sequelae cannot simply be side-stepped by dismissing NAFLD as pre-disease. Further research is required to quantify the associations of NAFLD with outcomes and to determine whether Wilson’s criteria for effective screening can be fulfilled [43], thereby informing the screening debate.

## Additional file

Additional file 1: **Table S1.** Characteristics of the primary-care databases included in the study. **Figure S1.** Identification of NAFLD patients in the IPCI database. **Table S2.** List of codes for the identification of NAFLD and description in ICD9CM, ICD10, Read Codes and SNOMEDCT US terminologies. **Table S3.** Number and proportion of patients with individual patient characteristic data available. **Table S4.** Point prevalence (95% CI) of NAFLD and NASH (per 100 persons) on 1 January of each calendar year in four primary-care databases, and pooled across databases. **Table S5.** One-year period prevalence (95% CI) of NAFLD (per 100 persons) on 1 January of each calendar year in four primary-care databases. **Table S6.** Point prevalence (95% CI) of NAFLD (per 1000 persons) by age categories and gender on 1 January 2015 in four primary-care databases. **Table S7.** Incidence estimates (95% CI) of NAFLD (per 1000 person-years) by calendar year in four primary-care databases, and pooled estimates across databases. **Table S8.** Incidence estimates (95% CI) of NAFLD (per 1000 person-years) in 2015 by age categories and gender in four primary-care databases. **Figure S2.** Pooled **a** prevalence (per 100 persons) and **b** incidence (per 1000 person-years) regressed over calendar year by meta-regression. **Figure S3.** Distribution of entry date for patients in the four databases. (DOCX 203 kb)

## Abbreviations

ALT: Alanine transaminase
ANOVA: Analysis of variance
AST: Aspartate transaminase
BMI: Body mass index
CI: Confidence interval
EHR: Electronic health record
EMIF: European Medical Information Framework
ERC: European Research Council
GP: General practitioner
HSD: Health Search Database
IPCI: Integrated Primary Care Information
NAFLD: Non-alcoholic fatty liver disease
NASH: Non-alcoholic steatohepatitis
NIHR: National Institute for Health Research
SIDIAP: Information System for Research in Primary Care
THIN: The Health Improvement Network
UK: United Kingdom
UMLS: Unified Medical Language System
US: United States
