# Supplementary material for: Real-world data reveal a diagnostic gap in non-alcoholic fatty liver disease
Source: BMC Med. 2018 Aug 13;16:130. doi: 10.1186/s12916-018-1103-x (PMC6088429; doi:10.1186/s12916-018-1103-x)

**Additional file 1**

**Supplementary Results**

**Table S1:** Characteristics of the primary care databases included in the study

| Characteristics | Health Search Database (HSD) | Integrated Primary Care Information (IPCI) | Information System for Research in Primary Care (SIDIAP) | The Health Improvement Network (THIN) |
| --- | --- | --- | --- | --- |
| Geographic origin of data | Italy | \|  \| The Netherlands \| \| --- \| --- \| | Catalonia region of Spain | \|  \| United Kingdom \| \| --- \| --- \| |
| Year of initial data collection | 1998 | 1995 | 2006 | 2002 |
| Terminology system(s) used to store diagnoses | ICD-9 | Free text, codes from Dutch College of General Practitioners, International Classification of Primary Care | ICD-10 | READ codes, free text |
| Enrolment | Covering 3.5% of Italy | Collective of GPs (typically 75-200 GPs) covering ~10% of the Netherlands | 279 primary care teams, covering 74% of Catalunia region of Spain | 562 general practices, covering 6.2% of the UK population |

**Figure S1:** Identification of NAFLD patients in the IPCI database

Table S2: List of codes for the identification of NAFLD and description in ICD9CM, ICD10, Read Codes, and SNOMEDCT US terminologies

| EVENT | UMLS CUI | UMLS Preferred String | Terminology Name | Terminology Code | Terminology String | Concept child included |
| --- | --- | --- | --- | --- | --- | --- |
| NAFLD_NASH | C0029546 | Other chronic nonalcoholic liver disease | ICD9CM | 571.8 | Chronic liver dis NEC | Y |
| NAFLD_NASH | C0029546 | Other chronic nonalcoholic liver disease | ICD9CM | 571.8 | Other chronic nonalcoholic liver disease | Y |
| NAFLD_NASH | C0029546 | Other chronic nonalcoholic liver disease | RCD | J61y. | Other non-alcoholic chronic liver disease | Y |
| NAFLD_NASH | C0029546 | Other chronic nonalcoholic liver disease | RCD | J61y. | Other nonalc.chronic liver dis | Y |
| NAFLD_NASH | C0029546 | Other chronic nonalcoholic liver disease | RCD | J61yz | Other non-alcoholic chronic liver disease NOS | Y |
| NAFLD_NASH | C0029546 | Other chronic nonalcoholic liver disease | RCD | J61yz | Other nonalc.chronic liver NOS | Y |
| NAFLD_NASH | C0029546 | Other chronic nonalcoholic liver disease | SNOMEDCT_US | 197314007 | Other non-alcoholic chronic liver disease | Y |
| NAFLD_NASH | C0029546 | Other chronic nonalcoholic liver disease | SNOMEDCT_US | 197314007 | Other non-alcoholic chronic liver disease (disorder) | Y |
| NAFLD_NASH | C0029546 | Other chronic nonalcoholic liver disease | SNOMEDCT_US | 197322000 | Other non-alcoholic chronic liver disease NOS | Y |
| NAFLD_NASH | C0029546 | Other chronic nonalcoholic liver disease | SNOMEDCT_US | 197322000 | Other non-alcoholic chronic liver disease NOS (disorder) | Y |
| NAFLD | C0400966 | Non-alcoholic Fatty Liver Disease | RCD | J61y1 | Non-alcoholic fatty liver | Y |
| NAFLD | C0400966 | Non-alcoholic Fatty Liver Disease | SNOMEDCT_US | 5360002 | Nonalcoholic fatty liver | Y |
| NAFLD | C0400966 | Non-alcoholic Fatty Liver Disease | SNOMEDCT_US | 5360002 | Nonalcoholic fatty liver (disorder) | Y |
| NAFLD | C0400966 | Non-alcoholic Fatty Liver Disease | SNOMEDCT_US | 197315008 | NAFLD - Nonalcoholic fatty liver disease | Y |
| NAFLD | C0400966 | Non-alcoholic Fatty Liver Disease | SNOMEDCT_US | 197315008 | Non-alcoholic fatty liver | Y |
| NAFLD | C0400966 | Non-alcoholic Fatty Liver Disease | SNOMEDCT_US | 197315008 | Non-alcoholic fatty liver (disorder) | Y |
| NAFLD | C0400966 | Non-alcoholic Fatty Liver Disease | SNOMEDCT_US | 371329005 | Nonalcoholic fatty liver | Y |
| NAFLD | C0400966 | Non-alcoholic Fatty Liver Disease | SNOMEDCT_US | 371329005 | Nonalcoholic fatty liver (disorder) | Y |
| NAFLD | C0494797 | Fatty (change of) liver, not elsewhere classified | ICD10 | K76.0 | Fatty (change of) liver, not elsewhere classified | Y |
| NAFLD |  |  | RCD | J61y900 | Fatty change of liver | Y |
| NAFLD |  |  | RCD | J61y911 | Fatty liver | Y |
| NASH | C0348750 | Other specified inflammatory liver diseases | ICD10 | K75.8 | Other specified inflammatory liver diseases | Y |
| NASH | C0348750 | Other specified inflammatory liver diseases | RCD | Jyu72 | [X]Oth specf inflamt liver dis | Y |
| NASH | C0348750 | Other specified inflammatory liver diseases | RCD | Jyu72 | [X]Other specified inflammatory liver diseases | Y |
| NASH | C0348750 | Other specified inflammatory liver diseases | RCDSY | Jyu72 | Oth specf inflamt liver dis | Y |
| NASH | C0348750 | Other specified inflammatory liver diseases | RCDSY | Jyu72 | Other specified inflammatory liver diseases | Y |
| NASH | C0348750 | Other specified inflammatory liver diseases | SNOMEDCT_US | 197554008 | [X]Other specified inflammatory liver diseases | Y |
| NASH | C0348750 | Other specified inflammatory liver diseases | SNOMEDCT_US | 197554008 | [X]Other specified inflammatory liver diseases (disorder) | Y |
| NASH | C2711227 | Steatohepatitis | RCD | J61y7 | Steatosis of liver | Y |
| NASH | C2711227 | Steatohepatitis | SNOMEDCT_US | 197321007 | Steatosis of liver | Y |
| NASH | C2711227 | Steatohepatitis | SNOMEDCT_US | 197321007 | Steatosis of liver (disorder) | Y |
| NASH | C2711227 | Steatohepatitis | SNOMEDCT_US | 442191002 | Steatohepatitis | Y |
| NASH | C2711227 | Steatohepatitis | SNOMEDCT_US | 442191002 | Steatohepatitis (disorder) | Y |
| NASH | C3241937 | Nonalcoholic Steatohepatitis | SNOMEDCT_US | 442685003 | NASH - Nonalcoholic steatohepatitis | Y |
| NASH | C3241937 | Nonalcoholic Steatohepatitis | SNOMEDCT_US | 442685003 | Nonalcoholic steatohepatitis | Y |
| NASH | C3241937 | Nonalcoholic Steatohepatitis | SNOMEDCT_US | 442685003 | Nonalcoholic steatohepatitis (disorder) | Y |
| NASH |  |  | RCD | J61y800 | Nonalcoholic steatohepatitis | Y |

UMLS: Unified Medical Language System; CUI: Concept Unique Identifier; ICD9CM: International Classification of Diseases – 9^th^ Revision – Clinical Modification; ICD10: International Classification of Diseases – 10^th^ Revision; RCD: Read Codes; SNOMEDCD_US: Systematized Nomenclature of Medicine--Clinical Terms Clinical Terms US version; Include Concept Child: Specifies whether the children of a UMLS concept are included in the list or the concept only; Y: Yes; N: No; NAFLD_NASH: NAFLD or NASH diagnosis.

Table S3: Number and proportion of patients with individual patient characteristic data available.

|  | HSD (n=24,027) | | IPCI (n=18,865) | | SIDIAP (n=77,107) | | THIN (n=23,385) | | Total (n=143,384) | |
| --- | --- | --- | --- | --- | --- | --- | --- | --- | --- | --- |
|  | n | % | n | % | n | % | n | % | N | % |
| Body Mass Index | 12,123 | 50.50% | 8,546 | 45.30% | 53,952 | 70.00% | 18,281 | 78.20% | 92,902 | 64.79% |
| History of diabetes or impaired fasting glucose | 4,332 | 18.00% | 3,862 | 20.50% | 15,420 | 20.00% | 4,909 | 21.00% | 28,523 | 19.89% |
| History of hypertension | 11,421 | 47.50% | 6,787 | 36.00% | 32,989 | 42.80% | 9,469 | 40.50% | 60,666 | 42.31% |
| Aspartate transaminase | 16,444 | 68.40% | 7,744 | 41.00% | 52,257 | 67.80% | 5,001 | 21.40% | 81,446 | 56.8% |
| Alanine transaminase | 17,416 | 72.50% | 12,678 | 67.20% | 66,561 | 86.30% | 19,109 | 81.70% | 115,764 | 80.74% |
| Platelet counts | 12,673 | 52.70% | 9,022 | 47.80% | 66,772 | 86.60% | 18,544 | 79.30% | 107,011 | 74.63% |
| AST to ALT ratio | 16,096 | 67.00% | 7,453 | 39.50% | 50,677 | 65.70% | 3,718 | 15.90% | 77,944 | 54.36% |
| FIB4 score | 10081 | 42.00% | 4,139 | 21.90% | 41,294 | 53.60% | 2,650 | 11.30% | 58,164 | 40.57% |

Table S4: Point-prevalence (95% CI) of NAFLD and NASH (per 100 persons) on the 1^st^ of January of each calendar year in four primary care databases, and pooled across databases

| Date  01/01/  Year | HSD database (Italy) | | | | IPCI database (Netherlands) | | | | SIDIAP database (Spain) | | | | THIN database (UK) | | | | Pooled point-prevalence (95% CI)  I2,  p-value of heterogeneity | |
| --- | --- | --- | --- | --- | --- | --- | --- | --- | --- | --- | --- | --- | --- | --- | --- | --- | --- | --- |
|  | Total | NAFLD | Prevalence | Total | | NAFLD | Prevalence | Total | | NAFLD | Prevalence | Total | | NAFLD | Prevalence |  | |  |
| 2004 | 995067 | 4947 | 0.50% | 50799 | | 313 | 0.62% | NA | | NA | NA | 3539620 | | 1864 | 0.05% | 0.39% (0.03%;0.75%)  99.95%, p<0.001 | |  |
| 2005 | 1017879 | 6334 | 0.62% | 57220 | | 434 | 0.76% | NA | | NA | NA | 3579021 | | 2501 | 0.07% | 0.48% (0.04%;0.933%)  99.96%, p<0.001 | |  |
| 2006 | 1042077 | 7989 | 0.77% | 33788 | | 385 | 1.14% | NA | | NA | NA | 3639293 | | 3195 | 0.09% | 0.66% (0.11%;1.21%)  99.97%, p<0.001 | |  |
| 2007 | 1067832 | 9998 | 0.94% | 74001 | | 823 | 1.11% | 4474843 | | 11052 | 0.25% | 3671770 | | 4114 | 0.11% | 0.60% (0.41%;0.79%)  99.97%, p<0.001 | |  |
| 2008 | 1076111 | 11852 | 1.10% | 198449 | | 2468 | 1.24% | 4517057 | | 14503 | 0.32% | 3691592 | | 5290 | 0.14% | 0.70% (0.46%;0.94%)  99.98%, p<0.001 | |  |
| 2009 | 1084335 | 13874 | 1.28% | 334507 | | 4614 | 1.38% | 4590082 | | 20165 | 0.44% | 3715133 | | 6716 | 0.18% | 0.82% (0.50%;1.13%)  99.98%, p<0.001 | |  |
| 2010 | 1093622 | 15954 | 1.46% | 464562 | | 7227 | 1.56% | 4598592 | | 27756 | 0.60% | 3659925 | | 8047 | 0.22% | 0.96% (0.55%;1.37%)  I2=99.99%, p<0.001 | |  |
| 2011 | 1095755 | 18009 | 1.64% | 534922 | | 9488 | 1.77% | 4547600 | | 36324 | 0.80% | 3600009 | | 9763 | 0.27% | 1.12% (0.61%;1.63%)  I2=99.99%, p<0.001 | |  |
| 2012 | 1094433 | 19844 | 1.81% | 715444 | | 14921 | 2.09% | 4484603 | | 44532 | 0.99% | 3557826 | | 11746 | 0.33% | 1.31% (0.68%;1.93%)  I2=99.99%, p<0.001 | |  |
| 2013 | 1085275 | 21503 | 1.98% | 795196 | | 19467 | 2.45% | 4430722 | | 54261 | 1.22% | 3492575 | | 13918 | 0.40% | 1.52% (0.77%;2.25%)  I2=99.99%, p<0.001 | |  |
| 2014 | 1068985 | 23001 | 2.15% | 775657 | | 19862 | 2.56% | 4358715 | | 64171 | 1.47% | 3235518 | | 15340 | 0.47% | 1.66% (0.83%;2.50%)  I2=99.99%, p<0.001 | |  |
| 2015 | 1034184 | 24035 | 2.32% | 875624 | | 24426 | 2.79% | 4277096 | | 74292 | 1.74% | 2899542 | | 15986 | 0.55% | 1.85% (0.91%;2.79%)  I2=99.99%, p<0.001 | |  |

Note: Pooled prevalence and heterogeneity estimates do not include SIDIAP for the years 2004-2006 inclusive.

Table S5: One-year period-prevalence (95% CI) of NAFLD (per 100 persons) on the 1^st^ of January of each calendar year in four primary care databases

| Year | HSD database (Italy) | | | IPCI database (Netherlands) | | | SIDIAP database (Spain) | | | THIN database (UK) | | | Pooled 1-year period prevalence (95% CI); I2; p-value of heterogeneity |
| --- | --- | --- | --- | --- | --- | --- | --- | --- | --- | --- | --- | --- | --- |
|  | Total | NAFLD | Prevalence (%), p-value* | Total | NAFLD | Prevalence (%) | Total | NAFLD | Total | Total | NAFLD | Prevalence (%) |  |
| 2004 | 1006502 | 1420 | 0.14%, <0.001 | 49404 | 86 | 0.17%, <0.001 | NA | NA | NA | 3559716 | 674 | 0.02%, , <0.001 | 0.11 (0.01; 0.21); 99.82%; p<0.001 |
| 2005 | 1029398 | 1772 | 0.17%, <0.001 | 47572 | 98 | 0.21%, <0.001 | NA | NA | NA | 3609900 | 762 | 0.02%, <0.001 | 0.13 (0.01; 0.25); 99.86%; p<0.001 |
| 2006 | 1054527 | 2154 | 0.20%, <0.001 | 54249 | 157 | 0.29%, <0.001 | NA | NA | NA | 3656466 | 996 | 0.03%, <0.001 | 0.17 (0.03; 0.32); 99.88%; p<0.001 |
| 2007 | 1071854 | 2040 | 0.19%, <0.001 | 130595 | 431 | 0.33%, <0.001 | 4493818 | 3673 | 0.08%, <0.001 | 3682664 | 1333 | 0.04%, <0.001 | 0.16 (0.11; 0.21); 99.86%; p<0.001 |
| 2008 | 1080343 | 2256 | 0.21%, <0.001 | 259426 | 816 | 0.31%, <0.001 | 4553336 | 5852 | 0.13%, <0.001 | 3703656 | 1592 | 0.04%, <0.001 | 0.17 (0.10; 0.25); 99.91%; p<0.001 |
| 2009 | 1088539 | 2405 | 0.22%, <0.001 | 397407 | 1357 | 0.34%, <0.001 | 4594410 | 7892 | 0.17%, <0.001 | 3688184 | 1794 | 0.05%, <0.001 | 0.19 (0.10; 0.29); 99.94%; p<0.001 |
| 2010 | 1094251 | 2421 | 0.22%, <0.001 | 514127 | 1641 | 0.32%, <0.001 | 4574616 | 9026 | 0.20%, <0.001 | 3630688 | 2030 | 0.06%, <0.001 | 0.20 (0.09; 0.30); 99.94%; p<0.001 |
| 2011 | 1094709 | 2290 | 0.21%, <0.001 | 627209 | 2416 | 0.39%, <0.001 | 4516128 | 8809 | 0.20%, <0.001 | 3579584 | 2438 | 0.07%, <0.001 | 0.21 (0.12; 0.31); 99.93%; p<0.001 |
| 2012 | 1089573 | 2244 | 0.21%, <0.001 | 772310 | 2934 | 0.38%, <0.001 | 4457615 | 10353 | 0.23%, <0.001 | 3525942 | 2824 | 0.08%, <0.001 | 0.22 (0.12; 0.33); 99.94%; p<0.001 |
| 2013 | 1076685 | 2237 | 0.21%, <0.001 | 793580 | 3103 | 0.39%, <0.001 | 4394772 | 10699 | 0.24%, <0.001 | 3364707 | 3178 | 0.09%, <0.001 | 0.23 (0.13; 0.34); 99.92%; p<0.001 |
| 2014 | 1051204 | 2078 | 0.20%, <0.001 | 838791 | 3695 | 0.44%, <0.001 | 4317926 | 11041 | 0.26%, <0.001 | 3068033 | 3234 | 0.11%, <0.001 | 0.25 (0.14; 0.36); 99.92%; p<0.001 |
| 2015 | 517092 | 1711 | 0.33%, Ref | 939592 | 4098 | 0.44%, Ref | 2138549 | 10885 | 0.51%, Ref | 2604230 | 3220 | 0.12%, Ref | 0.35 (0.13; 0.57); 99.96%; p<0.001 |

Table S6: Point-prevalence (95% CI) of NAFLD (per 1,000 persons) by age categories and gender on 1^st^ of January 2015 in four primary care databases

| Age category | HSD database (Italy) | | | | IPCI database (Netherlands) | | | | SIDIAP database (Spain) | | | | THIN database (UK) | | | |
| --- | --- | --- | --- | --- | --- | --- | --- | --- | --- | --- | --- | --- | --- | --- | --- | --- |
|  | Male prev. (%) | Female prev. (%) | Total prev. (%) | p-values* | Male prev. (%) | Female prev. (%) | Total prev. (%) | p-values | Male prev. (%) | Female prev. (%) | Total prev. (%) | p-values | Male prev. (%) | Female prev. (%) | Total prev. (%) | p-values |
| 18-29 y | 0.41% | 0.17% | 0.29% | <0.001 | 0.28% | 0.25% | 0.27% | <0.001 | 0.27% | 0.13% | 0.20% | <0.001 | 0.11% | 0.08% | 0.09% | <0.001 |
| 30-39 y | 1.34% | 0.38% | 0.85% | <0.001 | 1.26% | 0.73% | 0.99% | <0.001 | 0.77% | 0.33% | 0.55% | <0.001 | 0.38% | 0.18% | 0.28% | <0.001 |
| 40-49 y | 2.68% | 0.92% | 1.79% | <0.001 | 2.71% | 1.58% | 2.12% | <0.001 | 1.74% | 0.79% | 1.28% | <0.001 | 0.66% | 0.40% | 0.53% | <0.001 |
| 50-59 y | 3.73% | 2.09% | 2.90% | <0.001 | 4.00% | 3.41% | 3.70% | <0.001 | 2.85% | 2.22% | 2.53% | <0.001 | 0.92% | 0.82% | 0.87% | <0.001 |
| 60-69 y | 4.83% | 3.82% | 4.32% | Ref | 5.13% | 5.39% | 5.26% | Ref | 3.51% | 3.50% | 3.51% | Ref | 0.92% | 1.09% | 1.01% | Ref |
| 70-79 y | 4.71% | 4.03% | 4.35% | 0.715 | 4.84% | 5.61% | 5.25% | 0.874 | 2.77% | 3.25% | 3.03% | <0.001 | 0.67% | 0.81% | 0.74% | <0.001 |
| 80-89 y | 3.51% | 2.74% | 3.03% | <0.001 | 3.64% | 4.16% | 3.95% | <0.001 | 1.59% | 1.74% | 1.68% | <0.001 | 0.38% | 0.39% | 0.38% | <0.001 |
| ≥90 y | 1.57% | 1.21% | 1.31% | <0.001 | 1.89% | 2.06% | 2.02% | <0.001 | 0.63% | 0.58% | 0.59% | <0.001 | 0.15% | 0.10% | 0.12% | <0.001 |
| All age combined | 1.29% | 1.83% | 2.32% | <0.001 | 2.90% | 2.69% | 2.79% | <0.001 | 1.90% | 1.58% | 1.74% | <0.001 | 0.58% | 0.52% | 0.55% | <0.001 |

Y: years; Prev.: prevalence. P-values were estimated

Table S7: Incidence estimates (95% Confidence Intervals) of NAFLD (per 1,000 person-years) by calendar year in four primary care databases, and pooled estimates across databases.

| Year | HSD database (Italy) | | | IPCI database (Netherlands) | | | SIDIAP database (Spain) | | | THIN database (UK) | | | Pooled incidence  (95% CI) (per 1,000) | I2, p-value of heterogeneity |
| --- | --- | --- | --- | --- | --- | --- | --- | --- | --- | --- | --- | --- | --- | --- |
|  | Person-years | Events | Incidence (per 1,000); p-value* | Person-years | Events | Incidence (per 1,000) ; p-value* | Person-years | Events | Incidence (per 1,000) ; p-value* | Person-years | Events | Incidence (per 1,000) ; p-value* |  |  |
| 2004 | 1007121 | 1375 | 1.37; p<0.001 | 52759.93 | 81 | 1.54; p<0.001 |  |  |  | 3566526 | 604 | 0.17; p<0.001 | 1.02 (0.05: 1.98) | 99.81%, p<0.001 |
| 2005 | 1025792 | 1726 | 1.68; p:0.16 | 54897.57 | 87 | 1.58; p<0.001 |  |  |  | 3605379 | 680 | 0.19; p<0.001 | 1.15 (0.00: 2.34) | 99.86%, p<0.001 |
| 2006 | 1052222 | 2098 | 1.99; p<0.001 | 48326.12 | 90 | 1.86; p<0.001 |  |  |  | 3646374 | 907 | 0.25; p<0.001 | 1.36 (0.00: 2.76) | 99.88%, p<0.001 |
| 2007 | 1065058 | 1984 | 1.86; p<0.001 | 111216.5 | 265 | 2.38; p<0.001 | 4458077 | 3602 | 0.81; p<0.001 | 3672819 | 1206 | 0.33; p<0.001 | 1.32 (0.83: 1.82) | 99.85%, p<0.001 |
| 2008 | 1075802 | 2200 | 2.04; p<0.001 | 253169.7 | 692 | 2.73; p<0.001 | 4555564 | 5740 | 1.26; p<0.001 | 3701099 | 1449 | 0.39; p<0.001 | 1.60 (0.88: 2.33) | 99.91%, p<0.001 |
| 2009 | 1080457 | 2301 | 2.13; p<0.001 | 404424.8 | 1226 | 3.03; p<0.001 | 4584564 | 7770 | 1.69; p<0.001 | 3694249 | 1619 | 0.44; p<0.001 | 1.82 (0.87: 2.78) | 99.94%, p<0.001 |
| 2010 | 1085715 | 2333 | 2.15; p<0.001 | 502019.3 | 1460 | 2.91; p<0.001 | 4554139 | 8885 | 1.95; p<0.001 | 3605151 | 1826 | 0.51; p<0.001 | 1.88 (0.84: 2.92) | 99.94%, p<0.001 |
| 2011 | 1085780 | 2179 | 2.01; p<0.001 | 628868 | 2083 | 3.31; p<0.001 | 4468123 | 8678 | 1.94; p<0.001 | 3569132 | 2176 | 0.61; p<0.001 | 1.97 (0.98: 2.95) | 99.93%, p<0.001 |
| 2012 | 1084639 | 2127 | 1.96; p<0.001 | 780621.3 | 2726 | 3.49; p<0.001 | 4433242 | 10233 | 2.31; p<0.001 | 3533458 | 2532 | 0.72; p<0.001 | 2.12 (1.02: 3.22) | 99.94%, p<0.001 |
| 2013 | 1071698 | 2127 | 1.98; p<0.001 | 758426.7 | 2829 | 3.73; p<0.001 | 4338532 | 10595 | 2.44; p<0.001 | 3367287 | 2855 | 0.85; p<0.001 | 2.25 (1.15: 3.35) | 99.93%, p<0.001 |
| 2014 | 1054292 | 1948 | 1.85; p<0.001 | 826378 | 3330 | 4.03; p: 0.52 | 4251066 | 10944 | 2.57; p:0.25 | 3083252 | 2881 | 0.93; p<0.001 | 2.35 (1.23: 3.46) | 99.93%, p<0.001 |
| 2015 | 1007394 | 1614 | 1.60; Ref. | 940145.3 | 3847 | 4.09; Ref | 4149585 | 10850 | 2.61; Ref | 2609263 | 2819 | 1.08; Ref | 2.35 (1.29: 3.40) | 99.92%, p<0.001 |

Ref.: Reference category; *p-values are derived from Poisson models testing the effect of calendar year (sex and all age groups combined)

Table S8: Incidence estimates (95% CI) of NAFLD (per 1,000 person-years) in 2015 by age categories and gender in four primary care databases

| Age category (y) | HSD | | | | IPCI | | | | SIDIAP | | | | THIN | | | |
| --- | --- | --- | --- | --- | --- | --- | --- | --- | --- | --- | --- | --- | --- | --- | --- | --- |
|  | Male inc. (per 1,000) | Female inc. (per 1,000) | Total inc. (per 1,000) | p-values* | Male inc. (per 1,000) | Female inc. (per 1,000) | Total inc. (per 1,000) | p-value* | Male inc. (per 1,000) | Female inc. (per 1,000) | Total inc. (per 1,000) | p-value* | Male inc. (per 1,000) | Female inc. (per 1,000) | Total inc.(per 1,000) | p-value* |
| 18-29 | 0.54 | 0.19 | 0.37 | <0 | 0.87 | 0.64 | 0.76 | <0 | 0.62 | 0.27 | 0.44 | <0 | 0.33 | 0.22 | 0.28 | <0 |
|  |  |  |  | 0.001 |  |  |  | 0.001 |  |  |  | 0.001 |  |  |  | 0.001 |
| 30-39 | 1.13 | 0.31 | 0.71 | <0 | 2.57 | 1.67 | 2.11 | <0 | 1.38 | 0.71 | 1.05 | <0 | 0.87 | 0.61 | 0.74 | <0 |
|  |  |  |  | 0.001 |  |  |  | 0.001 |  |  |  | 0.001 |  |  |  | 0.001 |
| 40-49 | 1.93 | 0.83 | 1.37 | <0 | 4.19 | 3.37 | 3.76 | <0 | 2.56 | 1.72 | 2.15 | <0 | 1.3 | 1.05 | 1.17 | <0 |
|  |  |  |  | 0.001 |  |  |  | 0.001 |  |  |  | 0.001 |  |  |  | 0.001 |
| 50-59 | 2.56 | 2.03 | 2.29 | <0 | 5.59 | 6.27 | 5.94 | <0 | 3.85 | 4.45 | 4.15 | <0 | 1.67 | 1.87 | 1.77 | 0.142 |
|  |  |  |  | 0.001 |  |  |  | 0.001 |  |  |  | 0.001 |  |  |  |  |
| 60-69 | 3.12 | 3.08 | 3.1 | Ref. | 7.13 | 7.18 | 7.16 | Ref. | 4.85 | 5.19 | 5.03 | Ref. | 1.62 | 1.66 | 1.64 | Ref. |
| 70-79 | 3 | 2.06 | 2.49 | 0.005 | 5.48 | 6.62 | 6.08 | 0.002 | 3.43 | 4.11 | 3.8 | <0 | 1.1 | 1.26 | 1.19 | <0 |
|  |  |  |  |  |  |  |  |  |  |  |  | 0.001 |  |  |  | 0.001 |
| 80-89 | 1.2 | 0.87 | 0.99 | <0 | 4.44 | 4.18 | 4.28 | <0 | 1.51 | 1.69 | 1.62 | <0 | 0.46 | 0.67 | 0.58 | <0 |
|  |  |  |  | 0.001 |  |  |  | 0.001 |  |  |  | 0.001 |  |  |  | 0.001 |
| ≥90 | 0.58 | 0.11 | 0.24 | <0 | 1.98 | 0.37 | 0.8 | <0 | 0.57 | 0.24 | 0.33 | <0 | 0.12 | 0.05 | 0.07 | <0 |
|  |  |  |  | 0.001 |  |  |  | 0.001 |  |  |  | 0.001 |  |  |  | 0.001 |
| All ** | 1.92 | 1.3 | 1.6 | 0.001 | 4.14 | 4.04 | 4.09 | 0.42 | 2.68 | 2.56 | 2.61 | 0.016 | 1.1 | 1.06 | 1.08 | 0.244 |

Y: years; Inc: incidence; Ref.: Reference category; *p-values are derived from Poisson models testing the effect of age with Males and Females samples combined. Reference age category is 60-69 years old. ** p-values are derived from Poisson models testing the effect of sex with all ages combined.

Figure S2: Pooled a) prevalence (per 100 persons) and b) incidence (per 1,000 person years) regressed over calendar year by meta-regression

1. Prevalence


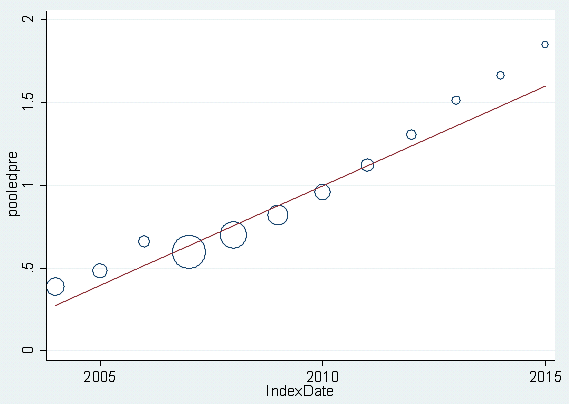


1. Incidence


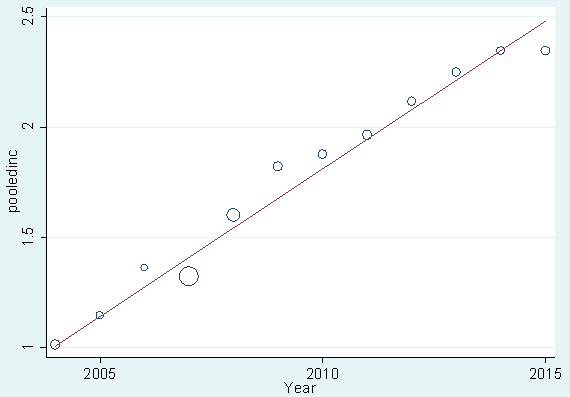


The linear effect of year was highly significant (p<0.001) in both models (estimates available on request).

Figure S3: Distribution of entry date for patients in the four databases

1. HSD


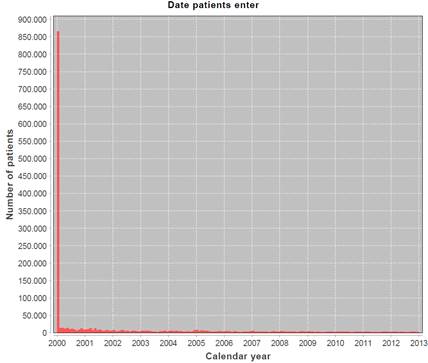


1. IPCI
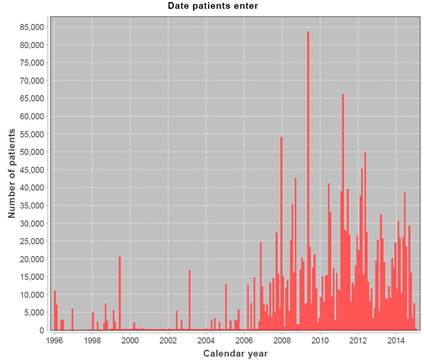

2. SIDIAP
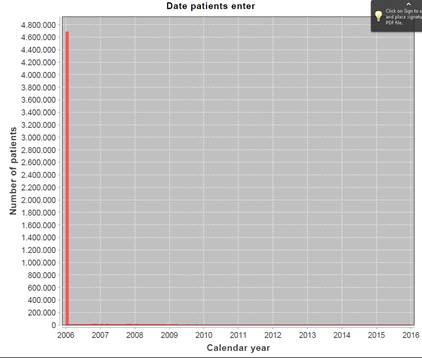

3. THIN
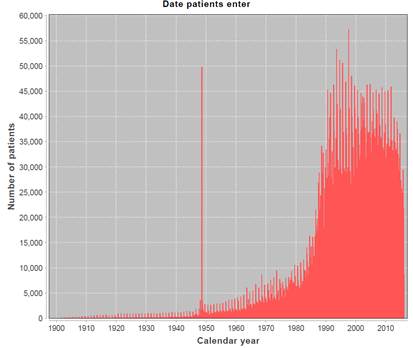

Supplement: Supplementary file 1 — Table S1. Characteristics of the primary-care databases included in the study. Figure S1. Identification of NAFLD patients in the IPCI database. Table S2. List of codes for the identification of NAFLD and description in ICD9CM, ICD10, Read Codes and SNOMEDCT US terminologies. Table S3. Number and proportion of patients with individual patient characteristic data available. Table S4. Point prevalence (95% CI) of NAFLD and NASH (per 100 persons) on 1 January of each calendar year in four primary-care databases, and pooled across databases. Table S5. One-year period prevalence (95% CI) of NAFLD (per 100 persons) on 1 January of each calendar year in four primary-care databases. Table S6. Point prevalence (95% CI) of NAFLD (per 1000 persons) by age categories and gender on 1 January 2015 in four primary-care databases. Table S7. Incidence estimates (95% CI) of NAFLD (per 1000 person-years) by calendar year in four primary-care databases, and pooled estimates across databases. Table S8. Incidence estimates (95% CI) of NAFLD (per 1000 person-years) in 2015 by age categories and gender in four primary-care databases. Figure S2. Pooled a prevalence (per 100 persons) and b incidence (per 1000 person-years) regressed over calendar year by meta-regression. Figure S3. Distribution of entry date for patients in the four databases. (DOCX 203 kb) [file 12916_2018_1103_MOESM1_ESM.docx]
